# Insulin Resistance Surrogates and Cognitive Impairment in Parkinson’s Disease: A Cross-Sectional Study with Interpretable Machine Learning

**DOI:** 10.3390/biomedicines14030493

**Published:** 2026-02-24

**Authors:** Hongming Liang, Yuru Jia, Hui Zhang, Danlei Wang, Haoheng Yu, Yongwen Yan, Jingyi Li, Liangkai Chen, Zheng Xue

**Affiliations:** 1 Department of General Practice, Tongji Hospital, Tongji Medical College, Huazhong University of Science and Technology, Wuhan 430030, China; d202482527@hust.edu.cn (H.L.); m202476631@hust.edu.cn (Y.J.); 2 Department of Neurology, The Second Affiliated Hospital of Guangxi Medical University, Nanning 530007, China; 3 Department of Rehabilitation Medicine, The First Affiliated Hospital of Guangxi Medical University, Nanning 530021, China; zh50322@sr.gxmu.edu.cn; 4 Department of Neurology, Tongji Hospital, Tongji Medical College, Huazhong University of Science and Technology, Wuhan 430030, China; wangdanlei10989@qiluhospital.com (D.W.); d202382302@hust.edu.cn (H.Y.); m202376603@hust.edu.cn (Y.Y.); 2022tj0093@hust.edu.cn (J.L.); 5 Department of Nutrition and Food Hygiene, Hubei Key Laboratory of Food Nutrition and Safety, School of Public Health, Tongji Medical College, Huazhong University of Science and Technology, Wuhan 430030, China; 6 Ministry of Education Key Lab of Environment and Health, School of Public Health, Tongji Medical College, Huazhong University of Science and Technology, Wuhan 430030, China

**Keywords:** insulin resistance, Parkinson’s disease, dementia, nomogram, machine learning, SHAP

## Abstract

**Background**: Insulin resistance (IR) has emerged as a key player in the pathogenesis of cognitive impairment in Parkinson’s disease (PD). This study aims to systematically compare glucolipotoxicity-based (TyG, AIP) versus adiposity-driven (TyG-BMI, METS-IR) IR indices for their associations with PD dementia and to develop a clinically applicable nomogram using an interpretable machine learning framework. **Methods**: This cross-sectional study analyzed 251 PD patients: 42 with normal cognition, 160 with mild cognitive impairment (PD-MCI) and 49 with dementia (PDD). Logistic and linear regression examined associations between IR indices and cognitive impairment across different domains. Six machine learning models were compared for dementia classification, with the optimal model interpreted using SHapley Additive exPlanations (SHAP) to construct a nomogram. **Results**: Each standard deviation increase in TyG and AIP was linked to 79% (OR 1.79, 95%CI 1.04–3.07) and 75% (OR 1.75, 95%CI 1.05–2.91) higher risk of PDD, respectively, but not PD-MCI. In contrast, TyG-BMI and METS-IR showed no significant associations with either condition. TyG showed linear negative correlations with memory and orientation, and inverted U-shaped associations with visuospatial function and attention. AIP exhibited linear negative correlation with memory. The logistic regression model achieved the highest performance (AUC of 0.759) among six machine learning models. Crucially, SHAP analysis visually quantified TyG as a top modifiable predictor, facilitating the construction of an interpretable clinical nomogram. **Conclusions**: Glucolipotoxicity-based indices (TyG, AIP), unlike BMI-dependent markers (TyG-BMI, METS-IR), are robustly linked to PD dementia through domain-specific linear or nonlinear patterns. This suggests metabolic dysregulation predicts risk independent of weight loss. Furthermore, integrating SHAP-based interpretability transforms complex algorithms into a transparent, actionable tool for early risk stratification.

## 1. Introduction

Parkinson’s disease (PD) ranks as the second most prevalent neurodegenerative disorder, affecting approximately 1.7% of individuals over 65 years, with a global burden exceeding 6.1 million cases—a figure that continues to rise with aging populations [1]. While the classical motor symptoms—tremor, rigidity, and bradykinesia—are well recognized, non-motor manifestations such as cognitive impairment are increasingly acknowledged for their substantial impact on quality of life. Cognitive dysfunction in PD, ranging from mild cognitive impairment (PD-MCI) to Parkinson’s disease dementia (PDD), affects approximately 40% of patients and is associated with faster disease progression and reduced functional independence [2].

Emerging evidence implicates insulin resistance (IR) in the pathophysiology of PD [3]. Mechanistically, IR is not limited to peripheral tissues but significantly impacts the brain [4]. Defective insulin signaling exacerbates α-synuclein aggregation and impairs autophagy [5,6], which specifically disrupts synaptic plasticity in cognitive circuits such as the hippocampus and prefrontal cortex rather than solely affecting motor pathways [7]. Furthermore, given that the brain is a lipid-rich organ, alterations in lipid metabolism—a core component of IR—play a crucial role. Dysregulated lipid homeostasis contributes to lipotoxicity, mitochondrial dysfunction, and oxidative stress, creating a pro-inflammatory environment that accelerates neurodegeneration [8,9]. Recent studies have reinforced the link between type 2 diabetes, IR, and an increased risk of PD dementia [10,11], highlighting the need to monitor metabolic status in this population.

Currently, the hyperinsulinemic-euglycemic clamp remains the gold standard for IR assessment but is invasive and impractical for clinical use [12]. The widely used Homeostatic Model Assessment for Insulin Resistance (HOMA-IR) also has limitations: it requires fasting insulin measurement, which is expensive, standardized poorly across laboratories, and not routinely performed in non-diabetic populations [13]. Consequently, there is a clinical need for accessible, non-invasive surrogate markers. Novel indices derived from routine fasting glucose and lipid profiles including the triglyceride-glucose (TyG) index and atherogenic index of plasma (AIP), as well as obesity-integrated indices like TyG-body mass index (TyG-BMI) and metabolic score for insulin resistance (METS-IR), have demonstrated comparable or even superior predictive value to HOMA-IR [14,15,16,17]. Despite their nature as indirect surrogates, these markers not only reflect IR but are also closely related to metabolic syndrome (MetS). Evidence from the Parkinson’s Progression Markers Initiative (PPMI) cohort indicates that PD patients with MetS exhibit more severe disease manifestations [18], suggesting that monitoring these composite biochemical parameters could offer valuable prognostic insights.

Although individual indices like TyG [19,20] and METS-IR [21] have been investigated in relation to PD onset or general cognitive function, few studies have systematically compared these four surrogate indices in the context of PD dementia. This comparison is clinically critical due to the unique phenotype of PD patients. Given that PD is frequently accompanied by weight loss and malnutrition [22], reliance on adiposity-driven IR indices (combining BMI, such as TyG-BMI and METS-IR) might introduce bias. It remains unclear whether glucolipotoxicity-based indices (such as TyG and AIP) offer superior predictive value over BMI-dependent markers in this specific population. Clarifying this distinction is essential for selecting the most appropriate screening tool.

Therefore, the objectives of this study are twofold. First, from an etiological perspective, we aim to systematically evaluate and compare the associations of these four IR indices with PD dementia and examine their domain-specific relationships with cognitive function. Second, from a translational predictive perspective, we aim to employ Least Absolute Shrinkage and Selection Operator (LASSO) regression and interpretable machine learning via SHapley Additive exPlanations (SHAP) to identify robust predictors and develop a transparent, data-driven nomogram. This dual approach seeks to clarify the metabolic contributions to PD dementia and provide a practical tool for personalized risk stratification.

## 2. Materials and Methods

### 2.1. Study Participants

This cross-sectional study was conducted in accordance with the Strengthening the Reporting of Observational studies in Epidemiology (STROBE) criteria (Supplementary Table S5). A total of 251 patients diagnosed with PD were consecutively enrolled from Tongji Hospital, Tongji Medical College, Huazhong University of Science and Technology, between January 2014 and March 2025. All participants met the Movement Disorder Society Clinical Diagnostic Criteria for PD [23].

Exclusion criteria were as follows: (1) history of stroke or other neurological disorders; (2) active malignancies or severe hepatic, renal, or cardiovascular diseases; (3) acute infectious diseases; (4) incomplete laboratory data required for IR index calculation and cognitive evaluation; (5) use of glucose- or lipid-lowering medications that could interfere with IR measurements, or medications known to impair cognition (e.g., anticholinergics, tricyclic antidepressants, benzodiazepines). Notably, patients with metabolic syndrome were included provided they were free of such pharmacological interference, ensuring a representative natural metabolic profile.

All clinical assessments were conducted by experienced neurologists during on state. Cognitive assessments were completed during the hospitalization or outpatient visit, typically within 48 h of blood sampling. Ethical approval was obtained from the Ethics Committee of Tongji Hospital. Given the retrospective nature of the study, informed consent was waived.

### 2.2. Calculation of IR Indices

Fasting blood samples were collected on the day of hospital admission or the following morning. Laboratory parameters included fasting blood glucose, triglycerides, and high-density lipoprotein cholesterol (HDL-C).

The following IR indices were computed using validated formulas [24,25], with higher values indicating greater degrees of IR. Logarithmic transformations are inherent to these formulas, designed to maximize the correlation with the gold-standard hyperinsulinemic-euglycemic clamp while normalizing the typically right-skewed distribution of metabolic data for linear analysis:

TyG = Ln [fasting triglycerides (mg/dL) × fasting glucose (mg/dL)/2].

AIP = Log_10_ (triglyceride/high-density lipoprotein cholesterol).

TyG-BMI = Ln [fasting triglycerides (mg/dL) × fasting glucose (mg/dL)/2] × BMI.

METS-IR = Ln [2  ×  fasting glucose (mg/dL)  +  triglycerides (mg/dL)]  ×  BMI/Ln [high-density lipoprotein cholesterol (mg/dL)].

### 2.3. Covariate Assessment

Comprehensive data were collected for all participants through structured interviews and clinical evaluations. Demographic characteristics included age, sex, educational attainment (categorized as below high school or high school and above), and marital status (married vs. unmarried). We considered several lifestyle factors, including smoking status (never, former, or current smoker), alcohol consumption (never, former, or current drinker), regular exercise (defined as engaging in moderate-to-vigorous physical activities at least three times a week), dietary preference (categorized as salty, bland, or moderate), and BMI. Categorization of specific variables (e.g., education, diet) was performed to capture relevant clinical thresholds and align with data collection instruments, while minimizing data dimensionality to ensure model stability.

PD-related clinical parameters were also considered, including duration of PD, levodopa equivalent daily dose, disease severity as assessed by the Hoehn and Yahr staging scale (from stage 1 to 5), and motor symptoms evaluated using the Movement Disorder Society-sponsored revision of the Unified Parkinson’s Disease Rating Scale III (UPDRS-III, 18 items with higher scores representing more severe movement impairment). Anxiety and depression symptoms were measured using the Hamilton anxiety rating scale (HAMA, 14 items) and Hamilton depression rating scale (HAMD, 24 items) respectively, where higher scores indicate greater symptom severity.

### 2.4. Outcome Assessment

Cognitive function was evaluated in all PD patients using the Montreal cognitive assessment (MoCA, score ranges from 0 to 30). For domain-specific analyses, cognitive performance was evaluated across six domains with the following score ranges: visuospatial function (0–5), language (0–6), attention (0–6), memory (0–5), executive function (0–3), and orientation (0–6). Higher scores reflect better cognitive performance. Based on established MoCA cutoffs and prior literature [26], the 251 patients were categorized into three cognitive status groups: 42 patients with normal cognition (PD-NC, MoCA > 25), 160 patients with mild cognitive impairment (PD-MCI, MoCA 21–25), and 49 patients with dementia (PDD, MoCA < 21).

### 2.5. Statistical Analysis

All statistical analyses were conducted using R software (version 4.4.1), SPSS (version 29) and Python (version 3.12.5). There were no missing data for the four IR indices or cognitive scores. Missingness among covariates did not exceed 20%. Continuous covariates were imputed with the median and categorical covariates with the mode. A two-tailed *p*-value < 0.05 was considered statistically significant. Continuous variables were expressed as median (interquartile range, IQR) and compared among the three groups using Kruskal–Wallis test, with Bonferroni-corrected post hoc pairwise comparisons when appropriate. Categorical variables were summarized as frequencies and percentages, and compared using the Chi-square test.

Multivariable logistic regression models were constructed to evaluate the associations of IR indices with the risk of PD-MCI and PDD, using the PD-NC group as the reference to establish a baseline of cognitively normal PD pathology and quantify the incremental metabolic risk associated with the transition to MCI and dementia. IR indices were categorized into quartiles (Q1–Q4), with the lowest quartile (Q1) serving as the reference category. The *p* for trend was calculated by entering the median value of each quartile as a continuous variable in the regression models. Two models were established to control for potential confounders while managing the risk of overfitting. Model 1 was unadjusted. For Model 2, to adhere to the Events Per Variable (EPV) principle [27] (which recommends approximately 10 outcome events per covariate) and given the limited sample size of the PDD group, we restricted adjustments to four key covariates: age, sex, educational level, and HAMA score. These covariates were consistently selected across LASSO analyses below and are well-established risk factors for cognitive decline in PD.

To verify the robustness of our findings, two sensitivity analyses were performed. First, IR indices were analyzed as continuous variables. To facilitate comparison across different indices, raw values were normalized to Z-scores based on their means and SD, and results are presented per 1-SD increase. Second, a supplementary sensitivity analysis using a fully adjusted model was conducted to account for a broader range of potential confounders, including covariates from model 2 plus BMI (excluded from TyG-BMI and METS-IR analyses to avoid multicollinearity) and all other clinical and lifestyle covariates described in Section 2.3.

To evaluate the associations between significant insulin resistance indices and six cognitive domains, multivariable linear regression models were constructed to examine associations with indices treated as both categorical quartiles (reference: Quartile 1) and continuous variables. Given the continuous nature of domain scores, full adjustment with all 17 covariates was performed. Robust (sandwich) variance estimators were applied to calculate standard errors and *p* values. *P* for trend was assessed using the median value of each quartile. Complementarily, to visualize dose–response relationships and test for non-linearity, restricted cubic spline (RCS) models were employed within the same fully adjusted regression framework. Likelihood ratio tests were used to evaluate the overall association (*P* for overall) and potential non-linearity (*P* for non-linearity).

### 2.6. Variables Selection Using LASSO Regression

To identify the most significant variables distinguishing PDD from non-dementia PD patients (comprising PD-NC and PD-MCI), we employed LASSO regression analysis [28]. This method is a highly effective statistical tool for variable selection and model simplification, particularly when dealing with high-dimensional data. In this study, all 17 clinical covariates and the 4 IR indices were included as independent variables, with PDD status as the dependent variable. We used 5-fold cross-validation to determine the optimal penalty coefficient (λ). During this process, we focused on two key λ values: λ_min_, which yields the minimum mean squared error, and λ_1se_, which corresponds to a more simplified model within one standard error of the minimum error. Ultimately, based on the λ_1se_ threshold, we retained five variables with non-zero coefficients, identifying them as significant variables for recognizing PDD.

### 2.7. Machine Learning Algorithms and Model Interpretation

To distinguish PDD from non-dementia PD patients, six machine learning models were developed: Logistic Regression (LR), Support Vector Machine (SVM), Multilayer Perceptron (MLP), LightGBM, XGBoost, and Random Forest. The models incorporated the five key features previously selected by LASSO regression. Hyperparameters for these models were optimized using Grid Search. Detailed descriptions of the models and their specific hyperparameters are presented in the Supplementary Table S4.

Given the limited sample size of the PDD group, we employed 5-fold cross-validation for model training and validation. Model performance was comprehensively evaluated using a range of metrics, including accuracy, sensitivity, precision, specificity, F1 Score, and AUC. Results are presented as mean ± SD. The Logistic Regression model was ultimately selected as the best performer.

To investigate the contribution of each feature to PDD, we utilized SHAP values derived from the logistic regression model. SHAP provides both local and global interpretability [29]. By calculating and visualizing the SHAP values, which represent the log-odds contribution of individual features, we gained insights into the risk factors associated with dementia in PD patients. Finally, the most important features were identified by ranking them based on their contributions to the final model.

### 2.8. Construction and Evaluation of Nomogram

To develop a clinically usable nomogram for PDD, previously selected five variables were used for intuitive scoring [30]. To address overfitting, internal validation was conducted via bootstrapping with 1000 resamples. The model’s performance was evaluated comprehensively: its discrimination was assessed using the area under the curve (AUC) of the ROC curve (with values ranging from 0.5 for no discrimination to 1 for perfect discrimination); its calibration was evaluated by creating calibration plots to compare predicted outcomes with observed ones; and its performance was assessed using decision curve analysis (DCA). Furthermore, we evaluated potential multicollinearity among the variables by calculating the variance inflation factor (VIF), where a VIF value greater than 5 was considered indicative of significant multicollinearity.

## 3. Results

### 3.1. Baseline Characteristics

Baseline analysis of the 251 patients (16.7% PD-NC, 63.7% PD-MCI, 19.5% PDD) revealed significant demographic and clinical distinctions (Table 1). Post hoc pairwise comparisons with Bonferroni correction demonstrated that patients in the PDD group were significantly older, had a higher proportion of females, and had a lower proportion of high school education compared to both PD-NC and PD-MCI groups. In addition, PDD group showed a significantly higher prevalence of hypertension compared to the PD-NC group. Furthermore, dietary patterns differed significantly, with the PDD group exhibiting a divergence toward extreme preferences (salty and bland) compared to the predominantly moderate diet observed in PD-MCI patients. Regarding clinical features, while no significant differences were observed in disease duration or levodopa equivalent daily dose, compared to PD-NC patients, both PD-MCI and PDD patients had higher UPDRS-III scores for motor impairment. Furthermore, the PDD group exhibited the most severe neuropsychiatric symptoms, with HAMA and HAMD scores significantly elevated compared to both PD-NC and PD-MCI patients. As anticipated, global cognitive function and all cognitive domain scores were significantly lower in the PDD group. Notably, several distinguishing features including age, sex, education, and HAMA scores, were subsequently confirmed as key predictors in the LASSO and SHAP analyses (see Section 3.4).

### 3.2. Association of IR Indices with PDD

After adjustment for covariates (Table 2), patients in the highest quartile (Q4) of both TyG (OR 5.21, 95% CI 1.18–23.08, *p* for trend = 0.032) and AIP (OR 4.36, 95% CI 1.03–18.46, *p* for trend = 0.031) exhibited a significantly increased risk of PDD compared to those in the lowest quartile (Q1). In sensitivity analyses treating the indices as continuous variables, each SD increase in the TyG (OR 1.79, 95% CI 1.04–3.07, *p* = 0.035) and AIP (OR 1.75, 95% CI 1.05–2.91, *p* = 0.031) was associated with a 79% and 75% higher risk of PDD, respectively. The consistency between quartile-based and continuous analyses underscores the robustness of these results, suggesting a stable dose–response relationship. Notably, neither the TyG-BMI index nor METS-IR showed a significant association with the risk of PDD. These results remained consistent in the fully adjusted model incorporating all 17 covariates (Supplementary Table S1).

Furthermore, no significant associations were observed between any of the four IR indices and the risk of PD-MCI. This lack of association implies that the impact of metabolic dysregulation on cognition may be stage-dependent, potentially exerting a threshold effect that becomes clinically evident only in the later stages of cognitive decline (i.e., dementia).

Taken together, these findings suggest a potential association between the TyG/AIP and the risk of PDD.

### 3.3. Association of TyG and AIP with Specific Cognitive Domains

**Figure 1:**
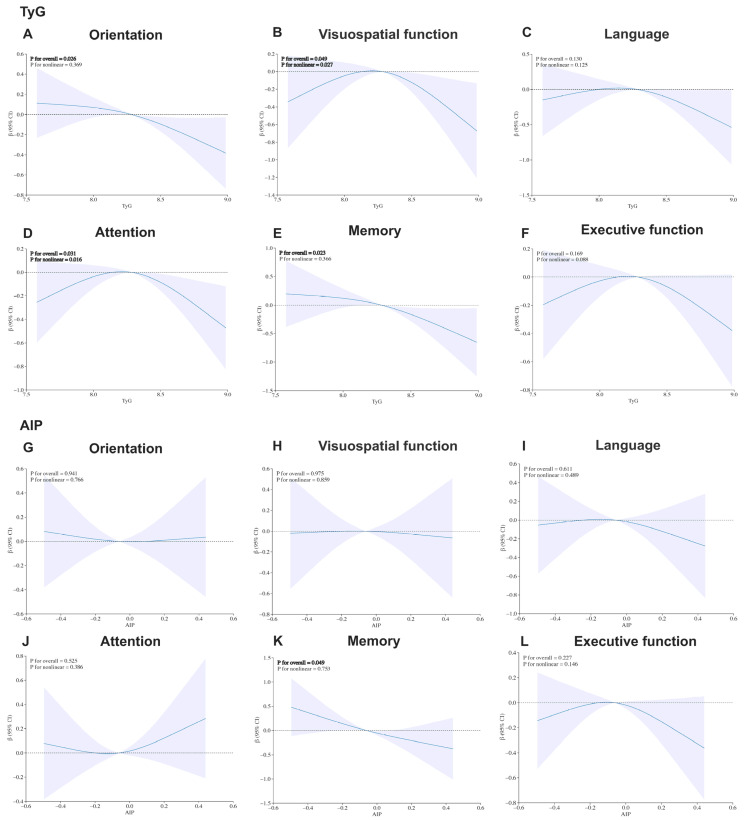
Restricted cubic spline curves of the associations between different cognitive domains and the TyG/AIP. (**A**–**F**) Restricted cubic spline curves showing dose–response relationships between the TyG index and six cognitive domains in Parkinson’s disease patients after full adjustment for all covariates (detailed in Section 2.5). (**G**–**L**) Dose–response relationships between the AIP and the corresponding six cognitive domains. Solid lines and shaded areas represent point estimates and 95% confidence intervals; the horizontal dashed line indicates β = 0. Regarding TyG, orientation and memory showed significant linear negative associations, while visuospatial function and attention exhibited inverted U-shaped patterns. For AIP, only a linear negative association with memory was observed.

As illustrated in Figure 1, the restricted cubic spline analyses revealed heterogeneous dose–response relationships between the TyG index and specific cognitive domains after full adjustment. Most notably, visuospatial function (*P* for overall = 0.049, *P* for non-linear = 0.027) and attention (*P* for overall = 0.031, *P* for non-linear = 0.016) displayed distinct inverted U-shaped patterns, with performance peaking at a TyG threshold of 8.3, suggesting a potential metabolic tipping point. In contrast, memory (*P* for overall = 0.023, *P* for non-linear = 0.366) and orientation (*P* for overall = 0.026, *P* for non-linear = 0.369) exhibited robust linear negative associations. Regarding AIP, a significant linear negative association was observed only with memory (*P* for overall = 0.049, *P* for non-linear = 0.753). These heterogeneous association patterns and specific effect sizes were further corroborated by the quartile-based linear regression analysis presented in Supplementary Table S2.

### 3.4. Construction and Evaluation of Machine Learning and Nomogram

**Figure 2:**
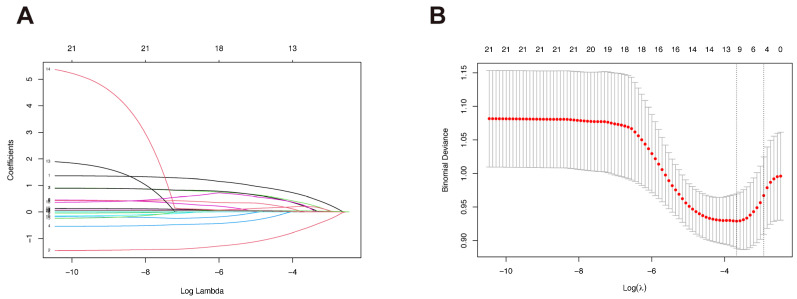
Feature selection using the LASSO regression model. (**A**) LASSO coefficient profiles of the 21 candidate features. Each line represents a variable; the trajectory shows how the coefficient shrinks to zero as the penalty parameter λ increases. (**B**) Selection of the optimal tuning parameter (λ) in the LASSO model. Vertical dashed lines indicate the minimum error (λ_min_) and the 1-standard error criterion (λ_1se_), where five non-zero coefficients were selected.

Based on LASSO regression analysis using the λ_1se_ threshold, we identified five independent risk factors: age, sex, education, HAMA, TyG (Figure 2A,B). We then employed these features to distinguish PDD from non- PDD patients using six machine learning algorithms. Among the evaluated models, Logistic Regression demonstrated the most proficient and comprehensive performance (Supplementary Table S3). It achieved the highest AUC (0.759 ± 0.070) and sensitivity (0.674 ± 0.143), indicating its superior ability to correctly identify PDD cases compared to other models. The Support Vector Machine (SVM) also showed comparable performance with an AUC of 0.752 ± 0.072 and a sensitivity of 0.633 ± 0.172. In contrast, while the Multilayer Perceptron (MLP), Random Forest, and LightGBM models achieved higher overall accuracy (ranging from roughly 0.79 to 0.80), they suffered from poor sensitivity (0.146, 0.384, and 0.323, respectively). This suggests that these models were biased towards the majority class (non-dementia), failing to effectively detect the minority PDD group. Therefore, considering the balance between sensitivity and specificity, Logistic Regression was selected as the optimal model for this study.

**Figure 3:**
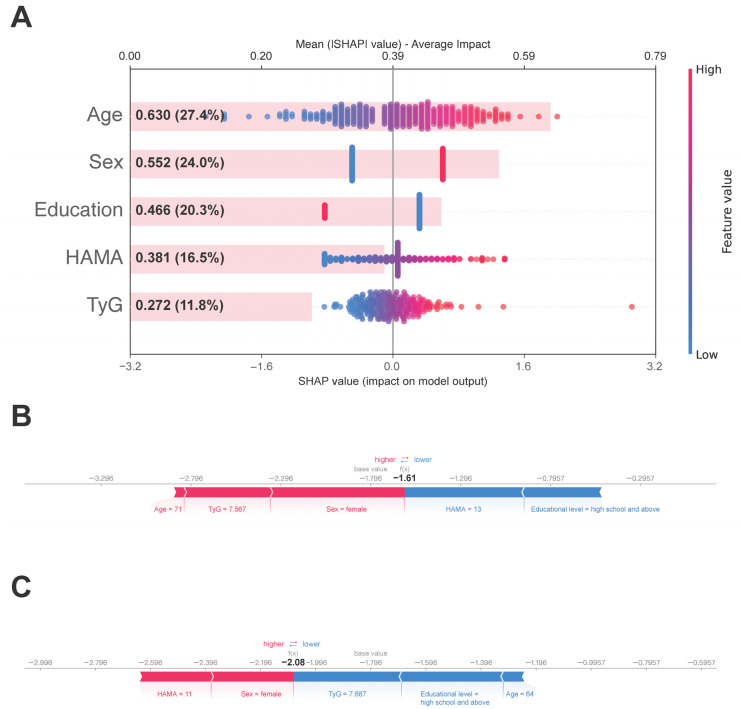
Interpretability analysis of the optimal Logistic Regression model using SHAP. (**A**) SHAP summary plot ranking the top five features by importance. Each dot represents a patient; red color indicates a high feature value, while blue indicates a low value. The *x*-axis represents the SHAP value (impact on model output). SHAP force plots illustrating individual predictions for representative patients with PDD (**B**) and without PDD (**C**).

To explore the contribution of each feature to PDD, we performed interpretability analysis on the Logistic Regression model using SHAP. The SHAP feature summary and importance plot (Figure 3A) showed that age had the largest contribution, accounting for 27.4% of the total, followed by sex (24.0%), educational level (20.3%), HAMA score (16.5%), TyG (11.8%). While TyG’s contribution is modest relative to demographic factors, it serves as a critical modifiable risk factor with specific translational significance. To further illustrate the direction and magnitude of these variables’ contributions to dementia risk, we separately presented the SHAP value distributions for two independent samples: one with PDD and one without (Figure 3B,C).

**Figure 4:**
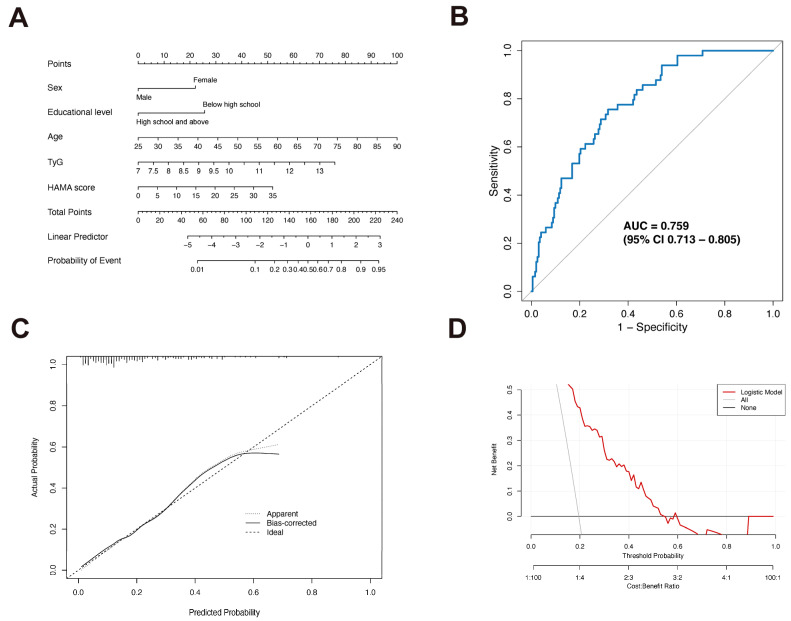
Construction and performance evaluation of the clinical nomogram. (**A**) Nomogram for identifying PDD risk integrating sex, educational level, age, TyG index, and HAMA score. (**B**) ROC curve analysis of the nomogram. (**C**) Calibration plot. The diagonal dotted line represents ideal prediction, while the solid line indicates the nomogram’s actual performance. (**D**) Decision Curve Analysis (DCA) demonstrating the net clinical benefit across threshold probabilities.

Finally, we constructed a nomogram based on these five variables (Figure 4A). The model’s performance was evaluated using multiple metrics: ROC curve analysis showed an AUC of 0.759 (95% CI: 0.713–0.805) (Figure 4B), indicating good discriminatory power. We performed internal validation using the Bootstrap method with 1000 resamples, which confirmed that the calibration curve showed good agreement with the ideal prediction curve, with a mean absolute error of 0.048 (Figure 4C). Decision curve analysis showed that the model has net benefit across probability thresholds from approximately 0.2 to 0.6 (Figure 4D). Furthermore, multicollinearity diagnostics showed low VIF values for all variables in the final model (sex: 1.048, educational level: 1.117, age: 1.018, TyG: 1.021, HAMA: 1.128), indicating the absence of significant multicollinearity among the variables and confirming that the regression estimates are stable and reliable.

## 4. Discussion

As the aging process accelerates, PD is progressively becoming a heavier social burden, with dementia further exacerbating this challenge. IR is recognized as one of the critical factors in the development of cognitive impairment. We systematically evaluated the associations between four novel IR indices and PD-related cognitive impairment. Both TyG and AIP were potentially associated with increased risk of PDD. Notably, TyG exhibited broader domain-specific associations, including linear negative correlations with memory and orientation, as well as inverted U-shaped associations with visuospatial function and attention while AIP was only linearly associated with memory decline. The machine learning analysis demonstrated that the Logistic Regression model can accurately and stably differentiate between patients with and without PDD. SHAP interpretability analysis identified the following as the most significant variables recognizing dementia: age, sex, educational level, HAMA score and TyG. Based on these findings, we developed a nomogram with an AUC of 0.759, demonstrating robust performance for potential use in clinical practice.

IR is typically characterized by hyperglycemia, dyslipidemia, hypertension, and obesity [31]. Various IR surrogate indices, derived from different combinations of these metabolic markers, can better reflect the extent of IR from different perspectives. Our results align with prior work examining the link between IR indices and cognitive impairment in general older populations. A longitudinal cohort study involving 4027 non-diabetic Chinese participants aged ≥ 45 years indicated that individuals with highest quartile levels of TyG index and Triglyceride-to-High-Density Lipoprotein Cholesterol ratio (TG/HDL-C, equivalent to AIP) had 24% and 34% increased risk of mild cognitive impairment, respectively, compared to those with lowest quartile levels [25]. In addition, other two population-based cross-sectional studies of 1352 and 1466 older Americans, respectively, reported significant negative associations of TyG index and AIP with cognitive function scores in older Americans [32,33]. Similarly, among population with PD, there is currently only one study that has collected data on the association between TyG index and PDD. This study, which enrolled 348 and 140 PD patients from two separate cohorts, suggested that the highest tertile of the TyG index is cross-sectionally associated with an increased PDD risk (OR 2.71, 95% CI 1.19–6.16) compared to the lowest tertile, though this association was not confirmed in the longitudinal analysis [34]. More recently, a cross-sectional study of 78 PD patients found that, when modeled as a continuous variable, the TyG index was independently associated with PDD risk (OR 7.520, 95% CI 1.147–49.324) [20]. Clinically, TyG and AIP offer practical advantages as screening tools. Both indices are derived from routine fasting laboratory tests, making them inexpensive, widely available, non-invasive, and initial triage tool to identify high-risk individuals who may benefit from more intensive diagnostic evaluation. Furthermore, incorporating these indices into PD care could help identify patients who might respond to metabolic interventions aimed at mitigating cognitive decline.

**Figure 5:**
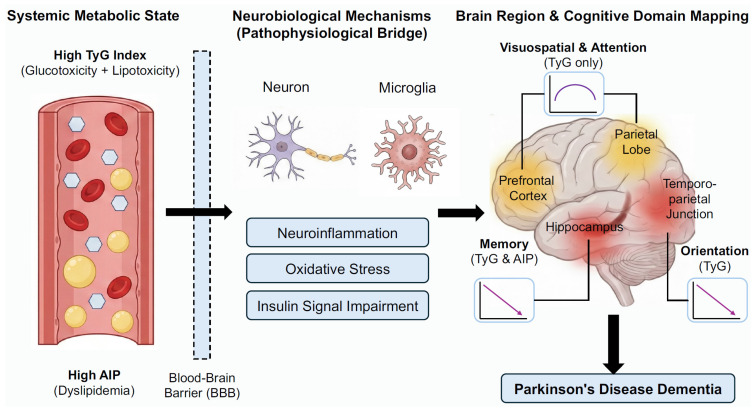
Schematic diagram illustrating the proposed contribution of insulin resistance indices to cognitive impairment in Parkinson’s disease. Systemic metabolic dysregulation, indicated by elevated TyG and AIP, may induce neuroinflammation, oxidative stress, and insulin signaling impairment in the brain. These pathological processes differentially affect specific brain regions.

Importantly, our study observed specific associations between IR indices and various cognitive domains in PD, with TyG index showed broader associations than AIP across cognitive domains (Figure 5). Specifically, both the TyG index and AIP were significantly linearly negatively associated with memory. The findings match those observed in previous studies pointing out that IR directly harming the hippocampus—a critical region for memory [35]. It is suggested to be induced through neuroinflammation, oxidative stress, and disrupted cerebral glucose metabolism [35,36]. Orientation deficits, observed only for TyG, may result from IR-induced dysfunction in temporoparietal networks crucial for spatial navigation and attentional control [37]. Most notably, we highlight the inverted U-shaped associations for visuospatial function and attention as key hypothesis-generating findings regarding metabolic resilience. This pattern offers new insights: before reaching the metabolic tipping point (TyG < 8.3), the brain might still be able to activate compensatory mechanisms to cope with metabolic challenges. However, once the IR level surpasses a critical threshold, its adverse effects on these specific cognitive domains may accelerate. This complex nonlinear relationship is not isolated. Similar findings have been reported in the general elderly population, where the TyG index exhibited an inverted J-shaped association with multiple cognitive functions, including memory, attention, verbal fluency, and executive function [38]. On the other hand, our study also did not find a significant link between the TyG index, AIP, and language or executive function. This should be interpreted with caution. Executive dysfunction is typically linked to prefrontal cortex function and is highly prevalent in PD-MCI patients, of which affecting about 20% [39]. This might suggest that this cognitive domain is less impacted by IR, or its effects might be mediated through alternative metabolic pathways not fully captured by TyG index or AIP. Therefore, we can infer that this unique nonlinear association between IR indices and different cognitive domains observed in PD patients might stem from several factors: differences in disease spectrum, characteristics of enrolled population, IR assessment methods, variations in established thresholds, and whether nonlinear models like restricted cubic splines were used for analysis. It is recommended that clinical PDD risk assessment should consider the specific characteristics of different cognitive domains and the particular thresholds of IR surrogates for a more precise and personalized risk evaluation.

Our study, using LASSO regression, identified four key clinical risk factors for PDD other than TyG: age, sex, educational level and anxiety, which aligns with previous findings. Age is one of the most widely recognized and strongest predictors for dementia. A cohort study including 760 PD patients showed that, compared to patients under 50, those aged 70 and above had a nearly threefold increased risk of developing dementia (HR 3.15) [40]. This accelerating effect of aging on cognitive decline is particularly pronounced in the later stages of the disease. Sex also plays an important role in the risk of cognitive impairment in PD, although findings are inconsistent. Our results suggest a higher risk of PDD in females, which is corroborated by the aforementioned cohort study from Taiwan [40]. However, another cohort study from Germany reported a higher dementia risk in male patients with PD (HR = 1.18), though this difference disappeared after considering the competing risk of mortality [41]. Beyond methodological differences, we speculate that this inconsistency may arise from the distinct impacts of racial genetics and environmental exposures, which warrants further investigation. Educational level is widely regarded as a key indicator of “cognitive reserve.” A meta-analysis incorporating 17 studies indicated that PD patients with a higher educational level not only had better overall cognitive function but also a 63% reduced risk of developing MCI compared to those with lower education [42]. This may explain why higher education serves as a protective factor, effectively delaying the clinical onset of cognitive decline. Furthermore, anxiety, a common non-motor symptom in PD, is closely associated with the development of PDD. Mounting evidence suggests that persistent anxiety may accelerate cognitive deterioration by affecting key brain networks (such as the default mode network) and neurotransmitter systems [43,44]. A retrospective study involving 118 patients with PD noted that those with anxiety symptoms scored significantly lower on cognitive domains, particularly attention, working memory, and language, and exhibited thinning of the bilateral anterior cingulate and left parietal cortex [45]. This could be because patients with anxiety allocate attentional resources to anxiety-inducing topics, thereby diverting resources and reducing the efficiency of cognitive control. Beyond these clinical determinants, we observed a distinct U-shaped dietary pattern. PD-MCI patients exhibited the highest adherence to a moderate diet, suggesting proactive lifestyle management, whereas PDD patients reverted to extreme preferences (salty and bland). This loss of dietary control likely reflects advanced pathology, where sensory deprivation prompts compensatory salt seeking [46], while dysphagia mandates bland, texture-modified regimens [47].

Our machine learning analysis revealed that among the six candidate models, the Logistic Regression algorithm had the most stable and superior performance for PDD, with AUC 0.759. While some machine learning studies utilizing small datasets report extremely high AUCs (>0.90), these often suffer from overfitting and lack external validity [48]. In our study, we prioritized generalizability over metric inflation. By strictly adhering to the EPV principle [27] and employing repeated stratified 5-fold cross-validation, our Logistic Regression model achieved a robust AUC of 0.759. This indicates that our model captures genuine clinical patterns rather than noise, making it a reliable tool for potential clinical application. Importantly, by employing SHAP analysis, we were able to provide interpretable insights into the contribution of key features. Specifically, SHAP analysis revealed that TyG ranks among the top five predictors, following established non-modifiable risk factors such as age, sex, and educational level. This finding is clinically significant: it demonstrates that TyG provides incremental predictive value beyond these traditional demographic factors. More importantly, unlike age or education, TyG is a modifiable metabolic risk factor, offering an actionable target for early intervention to potentially mitigate dementia risk. This enhances clinical transparency and trust in model interpretation. Similar approaches have been successfully applied in neurodegenerative disease research, where SHAP-based interpretability enabled the identification of metabolic and clinical biomarkers driving cognitive decline in both Alzheimer’s disease and PD cohorts [49]. Furthermore, our nomogram integrating machine learning–derived features demonstrated good calibration, echoing previous report that hybrid approaches combining machine learning-based variable selection with traditional clinical scoring systems can yield highly practical tools for bedside application [50]. Taken together, these results highlight the translational potential of interpretable machine learning frameworks not only in enhancing risk stratification for PDD but also in supporting personalized clinical decision-making.

Our study has several strengths, including the simultaneous evaluation of IR indices with distinct metabolic profiles, detailed cognitive domain analysis, and adjustment for multiple confounders. In addition, six machine learning algorithms were constructed and compared to recognize PDD. Finally, based on the SHAP interpretability framework, key factors were selected to construct a nomogram.

Limitations should also be acknowledged. Firstly, the cross-sectional design of this study limits our ability to establish a causal relationship between IR indices and cognitive impairment in PD patients. Longitudinal studies with larger samples are still needed in the future to further validate these findings. Secondly, to ensure the reliability of IR assessments, we strictly excluded patients using glucose- or lipid-lowering medications. While this rigorous exclusion prevents pharmacological interference and reflects the natural metabolic state (ensuring internal validity), we acknowledge that it may limit the external validity of our findings, as it excludes a segment of the PD population with treated metabolic comorbidities. Thirdly, despite comprehensive adjustment for numerous confounding factors and our best efforts to exclude medications that might influence the results, other potential confounders, such as genetics and environmental impact, may still affect the findings. Future research should further explore the differential effects of sporadic PD and genetic PD, as well as the potential impact of various hypoglycemic agents, lipid-lowering drugs, and cognitive-improving medications on IR and PDD interaction. Finally, this study was conducted in a single center, which may limit the generalizability of its findings to other populations, which deserved further validation from other centers.

## 5. Conclusions

In conclusion, our findings suggest that elevated TyG and AIP are associated with substantially higher dementia risk in PD (75–79% per SD increase) and exhibit distinct nonlinear metabolic thresholds (specifically TyG ≈ 8.3) for specific cognitive domains (particularly visuospatial function and attention). The SHAP-based interpretability framework successfully bridged the gap between complex machine learning algorithms and clinical application, providing a practical, transparent tool for early PDD risk stratification. These results underscore the potential role of metabolic dysregulation in PD cognitive decline and support targeting IR for preventive strategies. Future longitudinal, multicenter studies are warranted to validate these associations and evaluate the nomogram’s clinical utility.

## Figures and Tables

**Table 1:** Baseline characteristics of PD patients stratified by cognitive status.

Variable	Level	PD-NC (*N* = 42)	PD-MCI (*N* = 160)	PDD (*N* = 49)	Total (*N* = 251)	*p*
Number	n (%)	42 (16.7)	160 (63.7)	49 (19.5)	251 (100)	**-**
Female (%)	n (%)	13 (31.0)	75 (46.9)	33 (67.3) ^ab^	121 (48.2)	**0.002**
Age, year	Median (IQR)	54.5 (48.0–62.8)	61.0 (53.0–69.0) ^a^	65.0 (60.0–68.0) ^ab^	61.0 (53.0–68.0)	**<0.001**
BMI, kg/m^2^	Median (IQR)	23.0 (21.5–23.6)	23.0 (22.0–23.9)	23.0 (21.2–24.5)	23.0 (21.7–23.9)	0.457
Educational level, n (%)	High school and above	18 (42.9)	47 (29.4)	5 (10.2) ^ab^	70 (27.9)	**0.001**
Married	n (%)	35 (83.3%)	139 (86.9%)	42 (85.7%)	216 (86.1%)	0.838
Smoking status, n (%)	Never smoker	30 (71.4)	130 (81.2)	41 (83.7)	201 (80.1)	0.051
	Current smoker	8 (19.0)	18 (11.2)	1 (2.0)	27 (10.8)	
	Former smoker	4 (9.5)	12 (7.5)	7 (14.3)	23 (9.2)	
Alcohol intake, n (%)	Never drinker	29 (69.0)	132 (82.5)	42 (85.7)	203 (80.9)	0.057
	Current drinker	10 (23.8)	16 (10.0)	2 (4.1)	28 (11.2)	
	Former drinker	3 (7.1)	12 (7.5)	5 (10.2)	20 (8.0)	
Regular Exercise, n (%)	n (%)	20 (47.6)	48 (30.0)	21 (42.9)	89 (35.5)	0.053
Diet, n (%)	Salty	4 (9.5)	9 (5.6) ^a^	6 (12.2)b	19 (7.6)	**<0.001**
	Bland	6 (14.3)	2 (1.2) ^a^	7 (14.3)b	15 (6.0)	
	Moderate	32 (76.2)	149 (93.1) ^a^	36 (73.5)b	217 (86.5)	
Hypertension	n (%)	5 (11.9)	37 (23.1)	18 (36.7) ^a^	60 (23.9%)	**0.02**
Diabetes	n (%)	2 (4.8%)	18 (11.2%)	3 (6.1%)	23 (9.2%)	0.307
Total MoCA score	Median (IQR)	27.0 (27.0–28.0)	22.0 (21.0–23.0) ^a^	16.0 (13.0–18.0) ^ab^	21.0 (19.0–22.0)	**<0.001**
Visuospatial function	Median (IQR)	4.0 (4.0–5.0)	3.0 (2.0–3.0) ^a^	1.0 (0.0–2.0) ^ab^	3.0 (2.0–4.0)	**<0.001**
Language	Median (IQR)	6.0 (6.0–6.0)	5.0 (4.0–6.0) ^a^	4.0 (2.0–4.0) ^ab^	5.0 (4.0–6.0)	**<0.001**
Attention	Median (IQR)	6.0 (6.0–6.0)	5.0 (5.0–6.0) ^a^	5.0 (4.0–5.0) ^ab^	5.0 (5.0–6.0)	**<0.001**
Memory	Median (IQR)	4.0 (3.0–4.8)	3.0 (1.0–3.0) ^a^	0.0 (0.0–1.0) ^ab^	3.0 (1.0–3.0)	**<0.001**
Executive function	Median (IQR)	3.0 (3.0–3.0)	2.0 (1.0–3.0) ^a^	1.0 (0.0–2.0) ^ab^	2.0 (1.0–3.0)	**<0.001**
Orientation	Median (IQR)	6.0 (6.0–6.0)	6.0 (6.0–6.0) ^a^	5.0 (4.0–6.0) ^ab^	6.0 (6.0–6.0)	**<0.001**
Disease duration, years	Median (IQR)	3.0 (1.5–5.5)	3.0 (2.0–6.0)	4.0 (2.0–6.5)	3.0 (2.0–6.0)	0.609
Hoehn-Yahr stage, n (%)	Stage 1–2.5	33 (78.6%)	98 (61.2%)	27 (55.1%)	158 (62.9%)	0.053
	Stage 3–5	9 (21.4%)	62 (38.8%)	22 (44.9%)	93 (37.1%)	
UPDRS-III score	Median (IQR)	25.0 (8.2–37.8)	33.0 (18.0–45.2) ^a^	32.0 (21.0–49.0) ^a^	31.0 (18.0–45.0)	**0.025**
Levodopa equivalent daily dose, mg	Median (IQR)	418.7 (349.9–562.5)	425.0 (300.0–600.0)	400.0 (300.0–525.0)	424.9 (300.0–598.6)	0.587
HAMD score	Median (IQR)	11.0 (2.8–16.0)	16.0 (7.0–16.0) ^a^	16.0 (15.0–19.0) ^ab^	16.0 (8.5–16.0)	**<0.001**
HAMA score	Median (IQR)	9.0 (1.0–12.0)	13.0 (6.0–14.0) ^a^	13.0 (11.0–19.0) ^ab^	13.0 (7.0–14.0)	**<0.001**
Insulin resistance index	Median (IQR)					
TyG	Median (IQR)	8.1 (7.8–8.5)	8.3 (8.0–8.7)	8.5 (8.2–8.9) ^a^	8.3 (8.0–8.7)	**0.01**
AIP	Median (IQR)	−0.1 (−0.3–0.1)	−0.1 (−0.3–0.1)	0.0 (−0.2–0.2)	−0.1 (−0.3–0.1)	0.082
TyG-BMI	Median (IQR)	186.1 (172.7–203.1)	192.8 (177.2–207.6)	196.6 (175.9–210.6)	191.6 (176.2–207.4)	0.155
METS-IR	Median (IQR)	33.6 (29.3–35.7)	34.0 (30.7–37.4)	34.3 (30.6–38.4)	33.9 (30.6–37.4)	0.426

Note: Continuous variables are presented as Median (IQR); Categorical variables as n (%). *p* values indicate the overall difference among groups (Kruskal–Wallis test or Chi-square test). Bold values indicate statistical significance (*p* < 0.05). Post hoc pairwise comparisons (Bonferroni corrected): ^a^: *p* < 0.05 vs. PD-NC group. ^b^: *p* < 0.05 vs. PD-MCI group. Abbreviations: IQR: Interquartile Range; PD-NC: Parkinson’s Disease with Normal Cognition; PD-MCI: Parkinson’s Disease Mild Cognitive Impairment; PDD: Parkinson’s Disease Dementia; BMI: Body Mass Index; MoCA: Montreal Cognitive Assessment; UPDRS-III: Movement Disorder Society-Unified Parkinson’s Disease Rating Scale Part III; HAMD: Hamilton Depression Rating Scale; HAMA: Hamilton Anxiety Rating Scale; TyG: Triglyceride-Glucose Index; AIP: Atherogenic Index of Plasma.

**Table 2:** Associations of insulin resistance indices with MCI and Dementia risk in PD patients.

IR Index	Q1 (Reference)	Q2	Q3	Q4	*p* for Trend	Per SD Increment	*p*
TyG							
PD-MCI							
Model 1	1.00	1.28 (0.53, 3.07)	1.75 (0.69, 4.45)	2.31 (0.81, 6.59)	0.086	1.47 (0.97, 2.25)	0.072
Model 2	1.00	1.13 (0.42, 3.07)	1.14 (0.40, 3.26)	2.36 (0.73, 7.64)	0.199	1.43 (0.91, 2.24)	0.124
PDD							
Model 1	1.00	1.56 (0.47, 5.19)	2.50 (0.74, 8.45)	**5.94 (1.69, 20.86)**	**0.003**	**1.95 (1.20, 3.15)**	**0.007**
Model 2	1.00	1.36 (0.33, 5.59)	1.56 (0.37, 6.50)	**5.21 (1.18, 23.08)**	**0.032**	**1.79 (1.04, 3.07)**	**0.035**
AIP							
PD-MCI							
Model 1	1.00	1.08 (0.46, 2.54)	2.51 (0.88, 7.19)	1.66 (0.62, 4.42)	0.133	1.29 (0.90, 1.83)	0.166
Model 2	1.00	1.26 (0.47, 3.35)	2.72 (0.86, 8.56)	1.56 (0.52, 4.66)	0.209	1.32 (0.88, 1.97)	0.184
PDD							
Model 1	1.00	0.70 (0.21, 2.36)	**2.98 (0.92, 9.57)**	**3.50 (1.01, 12.18)**	**0.011**	**1.65 (1.08, 2.53)**	**0.022**
Model 2	1.00	1.08 (0.26, 4.42)	**3.10 (0.77, 12.47)**	**4.36 (1.03, 18.46)**	**0.031**	**1.75 (1.05, 2.91)**	**0.031**
TyG-BMI							
PD-MCI							
Model 1	1.00	1.08 (0.45, 2.58)	2.50 (0.91, 6.85)	2.22 (0.81, 6.12)	0.056	1.27 (0.87, 1.84)	0.215
Model 2	1.00	1.13 (0.42, 3.03)	2.40 (0.77, 7.47)	2.77 (0.89, 8.60)	0.059	1.30 (0.85, 2.00)	0.228
PDD							
Model 1	1.00	0.77 (0.25, 2.33)	1.69 (0.50, 5.68)	2.31 (0.71, 7.45)	0.085	1.46 (0.94, 2.25)	0.090
Model 2	1.00	0.98 (0.26, 3.73)	1.89 (0.45, 7.91)	3.87 (0.95, 15.71)	0.059	1.61 (0.97, 2.68)	0.067
METS-IR							
PD-MCI							
Model 1	1.00	1.03 (0.41, 2.56)	1.29 (0.50, 3.33)	1.50 (0.55, 4.07)	0.372	1.18 (0.82, 1.69)	0.377
Model 2	1.00	1.10 (0.40, 3.03)	1.40 (0.47, 4.13)	1.92 (0.63, 5.86)	0.226	1.29 (0.84, 1.98)	0.240
PDD							
Model 1	1.00	0.92 (0.29, 2.88)	1.10 (0.34, 3.55)	1.88 (0.58, 6.06)	0.272	1.36 (0.89, 2.07)	0.160
Model 2	1.00	1.33 (0.34, 5.25)	1.56 (0.38, 6.44)	3.94 (0.95, 16.25)	0.060	1.62 (0.93, 2.88)	0.054

Note: Values were presented as OR (95% CI). Bold values indicate statistical significance (*p* < 0.05). Abbreviations: IR: Insulin Resistance; Q1, Q2, Q3, Q4: Quartiles. Model 1 was unadjusted. Model 2 was adjusted for sex, age, educational level and HAMA score.

## Data Availability

The raw data supporting the conclusions of this article will be made available by the authors upon request.

## Supplementary Materials

The following supporting information can be downloaded at: https://www.mdpi.com/article/10.3390/biomedicines14030493/s1. Table S1: Associations of insulin resistance indices with MCI and Dementia risk in PD patients after fully adjusted; Table S2: Associations of TyG/AIP with different cognitive domains score; Table S3: Detailed performance metrics of various machine learning models for recognizing PDD patients; Table S4: Description and Optimized Hyperparameters of Machine Learning Models; Table S5: STROBE Statement—Checklist of items that should be included in reports of cohort studies.

## Abbreviations

The following abbreviations are used in this manuscript:

AIP	Atherogenic Index of Plasma
HAMA	Hamilton Anxiety Rating Scale
HAMD	Hamilton Depression Rating Scale
HOMA-IR	Model Assessment of Insulin Resistance
IR	Insulin Resistance
METS-IR	Metabolic Score for Insulin Resistance
MoCA	Montreal Cognitive Assessment
PD	Parkinson’s Disease
PD-MCI	Parkinson’s Disease with Mild Cognitive Impairment
PD-NC	Parkinson’s Disease with Normal Cognition
PDD	Parkinson’s Disease with Dementia
TyG	Triglyceride-Glucose Index
