# Supplementary material for: Insulin Resistance Surrogates and Cognitive Impairment in Parkinson’s Disease: A Cross-Sectional Study with Interpretable Machine Learning
Source: Biomedicines. 2026 Feb 24;14(3):493. doi: 10.3390/biomedicines14030493 (PMC13023561; doi:10.3390/biomedicines14030493)
Supplement: Supplementary file 1 [file biomedicines-14-00493-s001.zip › biomedicines-4140128-supplementary.pdf]

Insulin Resistance Surrogates and Cognitive Impairment in Parkinson's Disease: A Cross-Sectional Study with Interpretable Machine Learning

**Supplementary Table S1**	Associations of insulin resistance indices with MCI and Dementia risk in PD patients after fully adjusted
**Supplementary Table S2**	Associations of TyG/AIP with different cognitive domains score
**Supplementary Table S3**	Detailed performance metrics of various machine learning models for recognizing PDD patients
**Supplementary Table S4**	Description and Optimized Hyperparameters of Machine Learning Models.
**Supplementary Table S5**	STROBE Statement—Checklist of items that should be included in reports of cohort studies

Supplementary Table S1. Associations of insulin resistance indices with MCI and Dementia risk in PD patients after fully adjusted.

IR index	Q1	Q2	Q3	Q4	*p* for trend	Per SD increment	*p*
**TyG**							
**PD-MCI**	Reference	1.13 (0.39, 3.32)	1.35 (0.42, 4.32)	2.17 (0.60, 7.81)	0.245	1.50 (0.89, 2.52)	0.128
**PDD**	Reference	2.28 (0.50, 10.46)	1.64 (0.34, 7.98)	**7.19 (1.40, 37.00)**	**0.028**	**1.92 (1.04, 3.54)**	**0.036**
**AIP**							
**PD-MCI**	Reference	1.94 (0.66, 5.68)	3.48 (1.01, 11.99)	2.55 (0.73, 8.90)	0.066	1.50 (0.95, 2.37)	0.083
**PDD**	Reference	2.11 (0.48, 9.30)	4.16 (0.86, 20.16)	**5.26 (1.14, 24.25)**	**0.016**	**1.94 (1.09, 3.46)**	**0.025**
**TyG-BMI**							
**PD-MCI**	Reference	1.06 (0.36, 3.11)	2.36 (0.66, 8.47)	3.86 (0.79, 18.77)	0.056	1.26 (0.78, 2.03)	0.342
**PDD**	Reference	0.81 (0.19, 3.37)	1.40 (0.28, 6.88)	2.08 (0.27, 15.92)	0.446	1.7 (0.95, 3.03)	0.072
**METS-IR**							
**PD-MCI**	Reference	1.37 (0.47, 3.98)	1.56 (0.44, 5.56)	2.11 (0.53, 8.47)	0.293	1.29 (0.80, 2.07)	0.304
**PDD**	Reference	1.31 (0.33, 5.18)	1.01 (0.20, 5.15)	2.49 (0.40, 15.55)	0.445	1.73 (0.92, 3.28)	0.142

Values were presented as OR (95% CI)

Abbreviations: IR: Insulin Resistance; Q1, Q2, Q3, Q4: Quartiles.

Model was fully adjusted for sex, age, BMI (except for TyG-BMI and METS-IR), educational level, marital status, hypertension, diabetes, smoking status, alcohol intake, exercise, diet, disease duration, Hoehn-Yahr stage, UPDRS-III, levodopa prevalent dose, HAMA score, HAMD score.

Supplementary Table S2. Associations of TyG/AIP with different cognitive domains score

IR indices	Cognitive domain	Q2 vs Q1 β (95% CI), *p*	Q3 vs Q1 β (95% CI), *p*	Q4 vs Q1 β (95% CI), *p*	*p* for trend	Per SD increment β (95% CI), *p*
TyG	Visuospatial	0.38 (0.04, 0.72), **0.030**	0.10 (−0.43, 0.64), 0.701	−0.58 (−1.05, −0.11), **0.016**	0.041	−0.31 (−0.61, −0.01), **0.042**
	Language	0.23 (−0.25, 0.71), 0.342	0.14 (−0.35, 0.62), 0.587	−0.42 (−0.93, 0.09), 0.108	0.100	−0.19 (−0.47, 0.09), 0.181
	Attention	0.40 (0.05, 0.75), **0.025**	0.18 (−0.18, 0.54), 0.324	−0.52 (−1.01, −0.03), **0.038**	0.172	−0.12 (−0.39, 0.15), 0.382
	Memory	0.02 (−0.51, 0.54), 0.954	−0.49 (−1.04, 0.07), 0.091	−0.62 (−1.18, −0.07), **0.029**	**0.011**	−0.42 (−0.75, −0.10), **0.011**
	Executive function	0.30 (−0.08, 0.68), 0.123	0.23 (−0.15, 0.62), 0.237	−0.19 (−0.56, 0.19), 0.332	0.314	−0.02 (−0.23, 0.19), 0.850
	Orientation	−0.05 (−0.36, 0.26), 0.751	−0.11 (−0.43, 0.21), 0.498	−0.58 (−1.00, −0.16), **0.007**	**0.013**	−0.29 (−0.52, −0.06), **0.013**
AIP	Visuospatial	0.16 (−0.33, 0.64), 0.524	−0.13 (−0.67, 0.41), 0.642	−0.13 (−0.65, 0.39), 0.623	0.461	−0.19 (−0.84, 0.48), 0.582
	Language	0.12 (−0.37, 0.61), 0.624	0.18 (−0.33, 0.68), 0.491	−0.24 (−0.76, 0.28), 0.369	0.454	−0.32 (−0.97, 0.33), 0.333
	Attention	0.10 (−0.33, 0.54), 0.642	−0.16 (−0.62, 0.30), 0.499	0.11 (−0.33, 0.54), 0.628	0.900	0.11 (−0.46, 0.68), 0.712
	Memory	−0.21 (−0.75, 0.34), 0.463	−0.65 (−1.20, −0.10), **0.022**	−0.72 (−1.29, −0.16), **0.013**	**0.005**	−0.99 (−1.74, −0.24), **0.010**
	Executive function	0.28 (−0.09, 0.65), 0.141	0.12 (−0.27, 0.51), 0.552	−0.11 (−0.50, 0.28), 0.585	0.506	−0.27 (−0.76, 0.21), 0.266
	Orientation	0.12 (−0.25, 0.49), 0.533	−0.16 (−0.63, 0.31), 0.496	0.05 (−0.37, 0.47), 0.806	0.902	−0.17 (−0.74, 0.40), 0.560

Models were fully adjusted for sex, age, BMI, educational level, marital status, hypertension, diabetes, smoking status, alcohol intake, exercise, diet, disease duration, Hoehn-Yahr stage, UPDRS-III, Levodopa equivalent daily dose, HAMA score, HAMD score. Due to moderate skewness in certain cognitive domains, all analyses used robust (sandwich) variance estimators.

Supplementary Table S3. Detailed performance metrics of various machine learning models for recognizing PDD patients

Model	Accuracy	Sensitivity	Specificity	Precision	F1 Score	AUC
Logistic Regression	0.692 ± 0.051	0.674 ± 0.143	0.696 ± 0.065	0.351 ± 0.053	0.459 ± 0.071	0.759 ± 0.070
SVM	0.688 ± 0.066	0.633 ± 0.172	0.702 ± 0.085	0.345 ± 0.069	0.440 ± 0.088	0.752 ± 0.072
Random Forest	0.798 ± 0.052	0.384 ± 0.172	0.898 ± 0.065	0.482 ± 0.175	0.412 ± 0.151	0.719 ± 0.072
XGBoost	0.703 ± 0.078	0.601 ± 0.173	0.728 ± 0.107	0.358 ± 0.084	0.439 ± 0.099	0.721 ± 0.067
LightGBM	0.797 ± 0.034	0.323 ± 0.141	0.912 ± 0.042	0.491 ± 0.170	0.370 ± 0.127	0.704 ± 0.071
MLP	0.799 ± 0.039	0.146 ± 0.157	0.958 ± 0.066	0.320 ± 0.369	0.181 ± 0.188	0.759 ± 0.072

Values were presented as mean ± SD.

Supplementary Table S4. Description and Optimized Hyperparameters of Machine Learning Models.

Model	Description	Optimized Hyperparameters (via GridSearch)	Class Imbalance Strategy
Logistic Regression (LR)	A generalized linear model using a logistic function to estimate probabilities. Known for interpretability and robustness in small clinical datasets.	C=0.01 (Strong regularization); solver='lbfgs'; max_iter=1000	class_weight='balanced'
Support Vector Machine (SVM)	A supervised algorithm that finds an optimal hyperplane for classification. The RBF kernel was used to capture non-linear relationships.	kernel='rbf'; C=0.1; gamma=0.1; probability=True	class_weight='balanced'
Random Forest (RF)	An ensemble method constructing multiple decision trees. It reduces variance by averaging predictions from individual trees.	n_estimators=200; min_samples_leaf=4; max_depth=None	class_weight='balanced'
XGBoost	An efficient implementation of gradient boosting with depth-wise tree growth. It includes regularization terms to control overfitting.	n_estimators=50; max_depth=3; learning_rate=0.1	scale_pos_weight=4.12
LightGBM	A gradient boosting framework using leaf-wise tree growth. It is optimized for faster training speed and lower memory usage.	num_leaves=15; learning_rate=0.01; max_depth=-1	scale_pos_weight=4.12
Multilayer Perceptron (MLP)	A feedforward artificial neural network. A simplified architecture was chosen to accommodate the limited sample size.	hidden_layer_sizes=(50,); alpha=0.0001; learning_rate_init=0.01	None

Supplementary Table S5. STROBE Statement—Checklist of items that should be included in reports of *cohort studies*

Title: Insulin Resistance Surrogates and Cognitive Impairment in Parkinson's Disease: A Cross-Sectional Study with Interpretable Machine Learning

	Item No	Recommendation	Page No
**Title and abstract**	1	(*a*) Indicate the study's design with a commonly used term in the title or the abstract<br>(*b*) Provide in the abstract an informative and balanced summary of what was done and what was found	Pages 1 to 2 (Section: Abstract)
**Introduction**			
Background/rationale	2	Explain the scientific background and rationale for the investigation being reported	Introduction, paragraphs 1 to 3
Objectives	3	State specific objectives, including any prespecified hypotheses	Introduction, paragraph 3 and 4
**Methods**			
Study design	4	Present key elements of study design early in the paper	Methods, paragraph 1
Setting	5	Describe the setting, locations, and relevant dates, including periods of recruitment, exposure, follow-up, and data collection	Methods, paragraphs 1 to 12
Participants	6	(*a*) Give the eligibility criteria, and the sources and methods of selection of participants. Describe methods of follow-up<br>(*b*) For matched studies, give matching criteria and number of exposed and unexposed	Methods, paragraph 1 to 3<br>N/A
Variables	7	Clearly define all outcomes, exposures, predictors, potential confounders, and effect modifiers. Give diagnostic criteria, if applicable	Methods, paragraphs 4 to 12
Data sources/ measurement	8*	For each variable of interest, give sources of data and details of methods of assessment (measurement). Describe comparability of assessment methods if there is more than one group	Methods, paragraphs 4 to 12
Bias	9	Describe any efforts to address potential sources of bias	Methods, paragraph 1 to 3
Study size	10	Explain how the study size was arrived at	Methods, paragraph 1

Quantitative variables	11	Explain how quantitative variables were handled in the analyses. If applicable, describe which groupings were chosen and why	Methods, paragraph 13
Statistical methods	12	(*a*) Describe all statistical methods, including those used to control for confounding	Methods, paragraph 13-18
		(*b*) Describe any methods used to examine subgroups and interactions	N/A
		(*c*) Explain how missing data were addressed	Methods, paragraph 1
		(*d*) If applicable, explain how loss to follow-up was addressed	N/A
		(*e*) Describe any sensitivity analyses	Methods, paragraph 17, training and testing sets
**Results**			
Participants	13*	(a) Report numbers of individuals at each stage of study—eg numbers potentially eligible, examined for eligibility, confirmed eligible, included in the study, completing follow-up, and analysed	Results, paragraphs 1; Table 1
		(b) Give reasons for non-participation at each stage	N/A
		(c) Consider use of a flow diagram	Flowchart (Figure 1)
Descriptive data	14*	(a) Give characteristics of study participants (eg demographic, clinical, social) and information on exposures and potential confounders	Results, paragraph 1; Table 1
		(b) Indicate number of participants with missing data for each variable of interest	N/A
		(c) Summarise follow-up time (eg, average and total amount)	N/A
Outcome data	15*	Report numbers of outcome events or summary measures over time	Results, paragraph 1; Table 1
Main results	16	(*a*) Give unadjusted estimates and, if applicable, confounder-adjusted estimates and their precision (eg, 95% confidence interval). Make clear which confounders were adjusted for and why they were included	Results, paragraphs 2 to 3; Table 2
		(*b*) Report category boundaries when continuous variables were categorized	N/A
		(*c*) If relevant, consider translating estimates of relative risk into absolute risk for a meaningful time period	N/A
Other analyses	17	Report other analyses done—eg analyses of subgroups and interactions, and sensitivity analyses	Results, paragraph 3-6; Table 3, Figure 1

Discussion

Key results	18	Summarise key results with reference to study objectives	Discussion, paragraph 1
Limitations	19	Discuss limitations of the study, taking into account sources of potential bias or imprecision. Discuss both direction and magnitude of any potential bias	Discussion, paragraph 7
Interpretation	20	Give a cautious overall interpretation of results considering objectives, limitations, multiplicity of analyses, results from similar studies, and other relevant evidence	Discussion, paragraphs 2 to 5
Generalisability	21	Discuss the generalisability (external validity) of the study results	Discussion, paragraph 5-6
**Other information**			
Funding	22	Give the source of funding and the role of the funders for the present study and, if applicable, for the original study on which the present article is based	Funding, page 23

*Give information separately for cases and controls in case-control studies and, if applicable, for exposed and unexposed groups in cohort and cross-sectional studies.

Note: An Explanation and Elaboration article discusses each checklist item and gives methodological background and published examples of transparent reporting. The STROBE checklist is best used in conjunction with this article (freely available on the Web sites of PLoS Medicine at http://www.plosmedicine.org/, Annals of Internal Medicine at http://www.annals.org/, and Epidemiology at http://www.epidem.com/). Information on the STROBE Initiative is available at http://www.strobe-statement.org.
